# The Curious Case of Concomitant Hypo-Hyperdontia: A Case Report

**DOI:** 10.7759/cureus.70942

**Published:** 2024-10-06

**Authors:** Tanya Prasad, Yusuf Ronad, Renuka Pawar, Chanamallappa Ganiger, Sandesh Phaphe, Pratap Mane, Seema Patil

**Affiliations:** 1 Department of Orthodontics and Dentofacial Orthopedics, School of Dental Sciences, Krishna Vishwa Vidyapeeth, Karad, IND

**Keywords:** canine substitution, concomitant hypo-hyperdontia, dentistry, hyperdontia, hypodontia, mesiodens, missing lateral incisors, orthodontics, supernumerary teeth

## Abstract

Amongst the spectrum of dental developmental anomalies, hypodontia is a condition characterized by congenitally missing teeth. Whereas, hyperdontia is a condition that results in the development of an excessive number of teeth. These two conditions are regarded as polar opposites in the progression of dentition development. Their simultaneous occurrence in a patient results in a rare phenomenon known as concomitant hypo-hyperdontia (CHH). This report presents a case of CHH highlighting the importance of diagnosis and adequate treatment planning in achieving a balanced and esthetic smile.

## Introduction

​​Hypodontia is among the most frequent developmental abnormalities and is defined by the congenital absence of one or more teeth. The reported prevalence of hypodontia has been reported to be 1.6-9.6% [1]. Excluding the third molars, the most commonly missing teeth are mandibular second premolars followed by maxillary lateral incisors and maxillary second premolars [2]. 

Another frequently observed disturbance of odontogenesis is hyperdontia which is defined as the presence of an excess number of teeth [3]. A common example of hyperdontia is the mesiodens which occurs when a supernumerary tooth erupts between the two central incisors. It has a reported prevalence of 82% in the maxilla with 0.15-3.8% occurrence in the permanent dentition [4]. The mesiodens can have a conical crown or have a shape similar to that of a natural tooth. 

Concomitant hypo-hyperdontia (CHH) is an exceptionally rare condition characterized by the simultaneous presence of both hypodontia and hyperdontia in a single individual. The term was introduced by Gibson in 1979, who classified the condition according to the distribution of these anomalies. Its reported prevalence ranges from 0.002% to 3.1%, with no significant gender-based differences in occurrence [5].

There are few case reports exploring the occurrence of this condition and detailing its management. This report describes the interdisciplinary management of one such case. 

## Case presentation

Case history 

**Figure 1:**
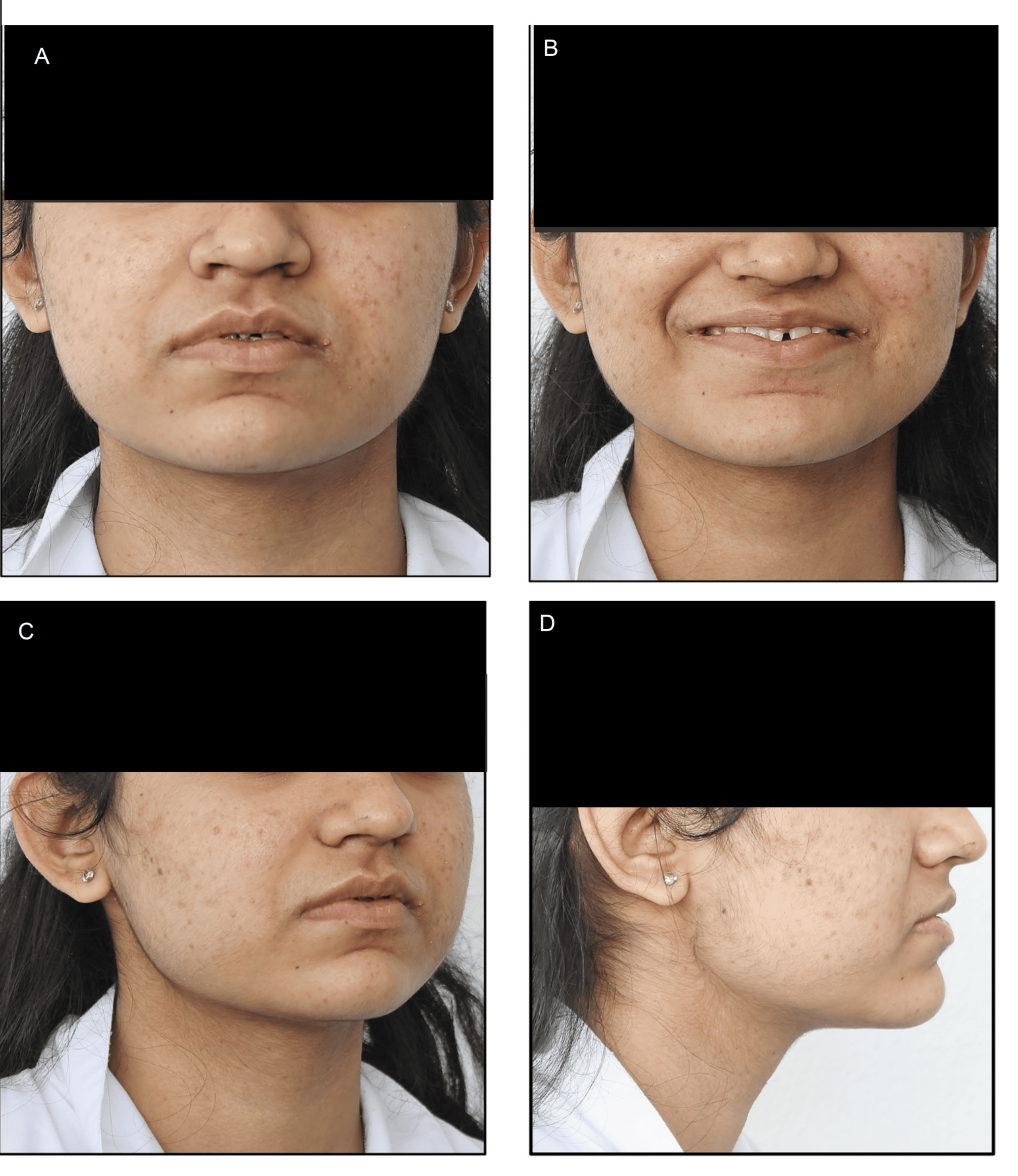
Extraoral pretreatment photographs: (A) Frontal, (B) Smiling, (C) 3/4th, and (D) Lateral

An 18-year-old female patient reported to the Department of Orthodontics and Dentofacial Orthopedics with the primary concern of spacing and an extra tooth between the upper front teeth. There was no family history of hereditary missing or supernumerary teeth. On extraoral examination, a mesoprosopic facial morphology was seen with a mildly straight facial profile. The clinical Frankfort-mandibular plane angle (FMA) and nasolabial angle were average. Lips were potentially competent with a deep mentolabial sulcus (Figure 1).

**Figure 2:**
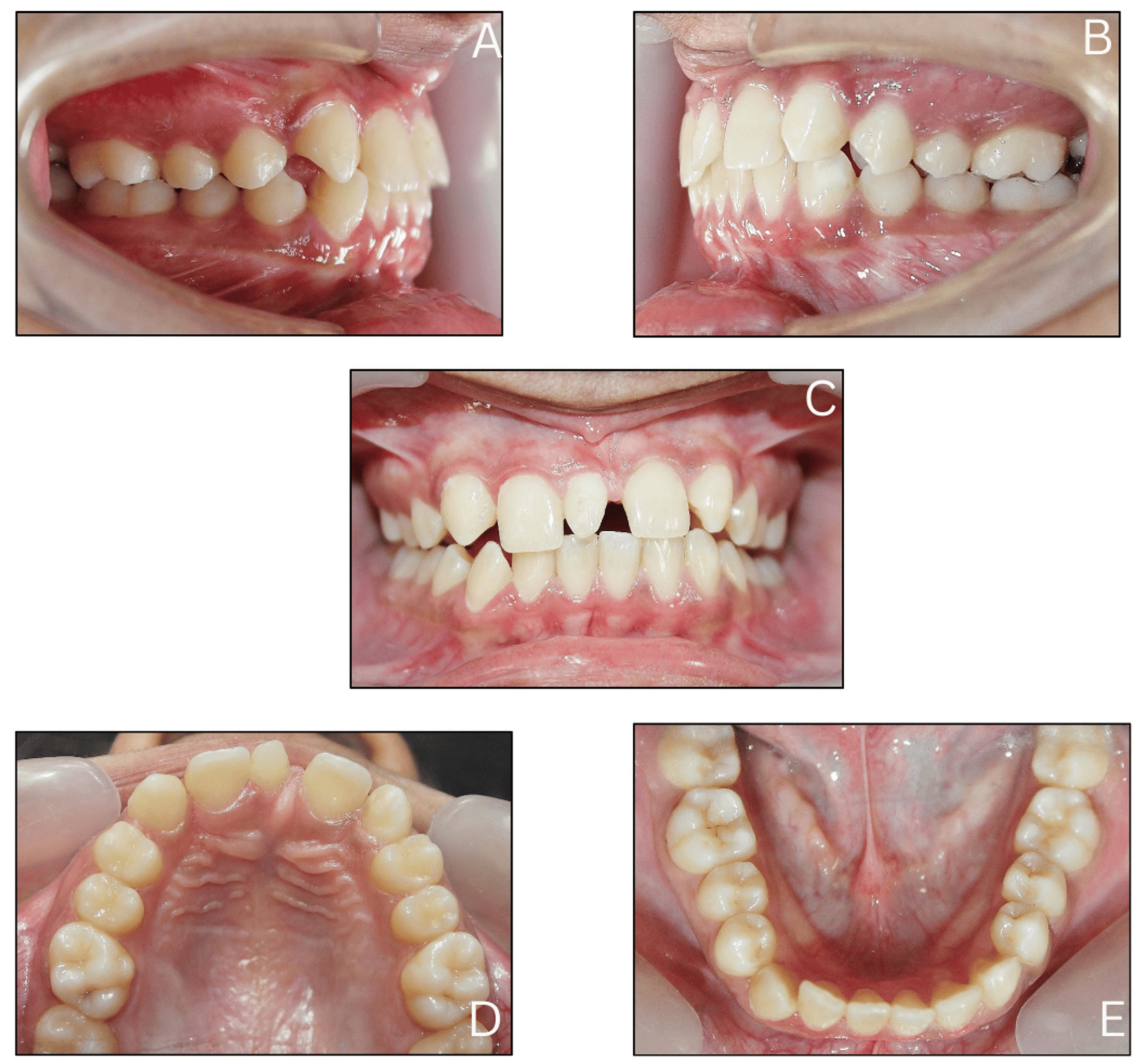
Intraoral pretreatment photographs: (A) Right lateral, (B) Left lateral, (C) Frontal, (D) Maxillary occlusal, and (E) Mandibular occlusal

Intraoral examination disclosed the presence of a conical supernumerary tooth between the central incisors. The bilateral absence of lateral incisors was also noted. The canines showed a bilateral Angle Class II relationship. The molar relation was Class I on the right side and Class II on the left side, classifying as Angle's Class II subdivision. Spacing was observed in the upper anteriors with mild crowding in the lower anteriors (Figure 2).

Radiographic examination 

**Figure 3:**
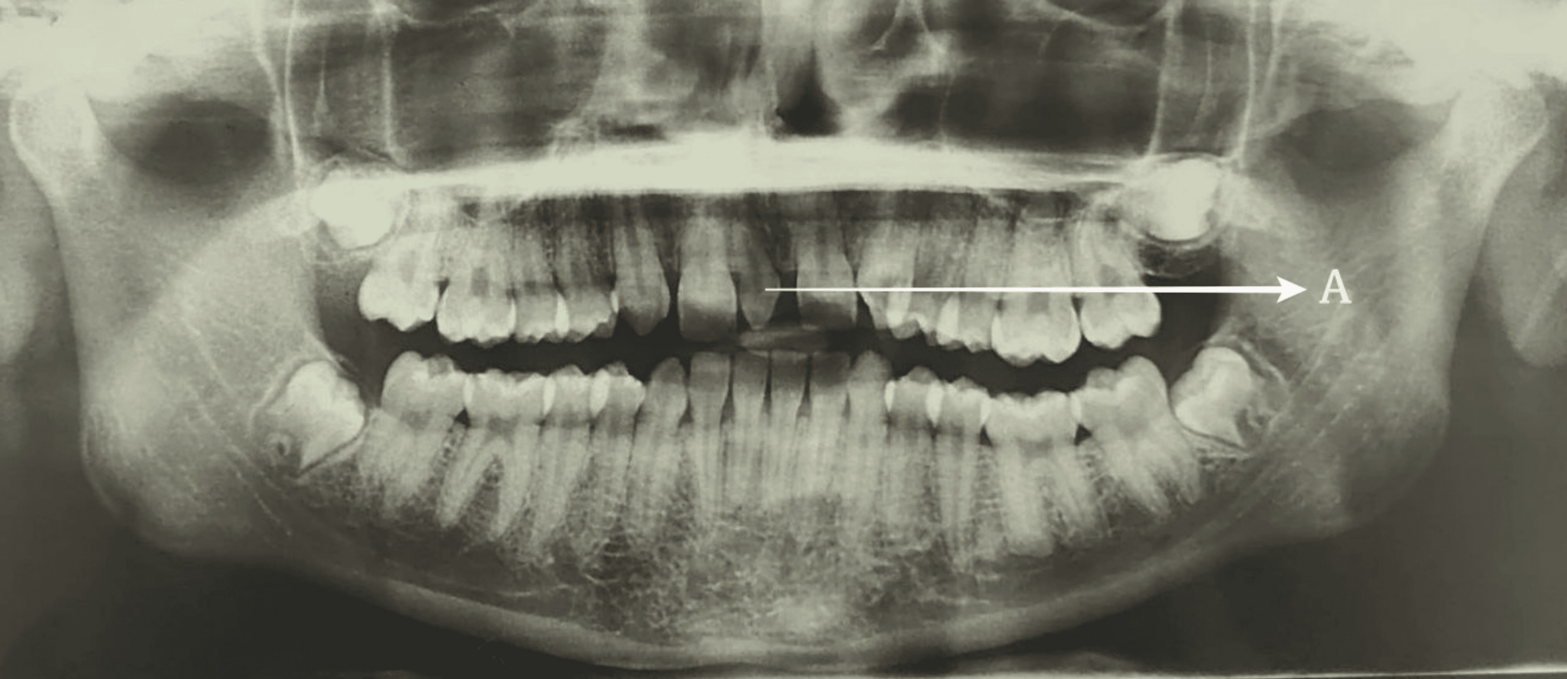
Pretreatment orthopantomography showing the presence of mesiodens (A) between 11-21

A panoramic radiograph confirmed the absence of lateral incisors bilaterally and the presence of a vertically oriented conical-shaped mesiodens which was smaller in dimension as compared to the normal teeth (Figure 3).

**Figure 4:**
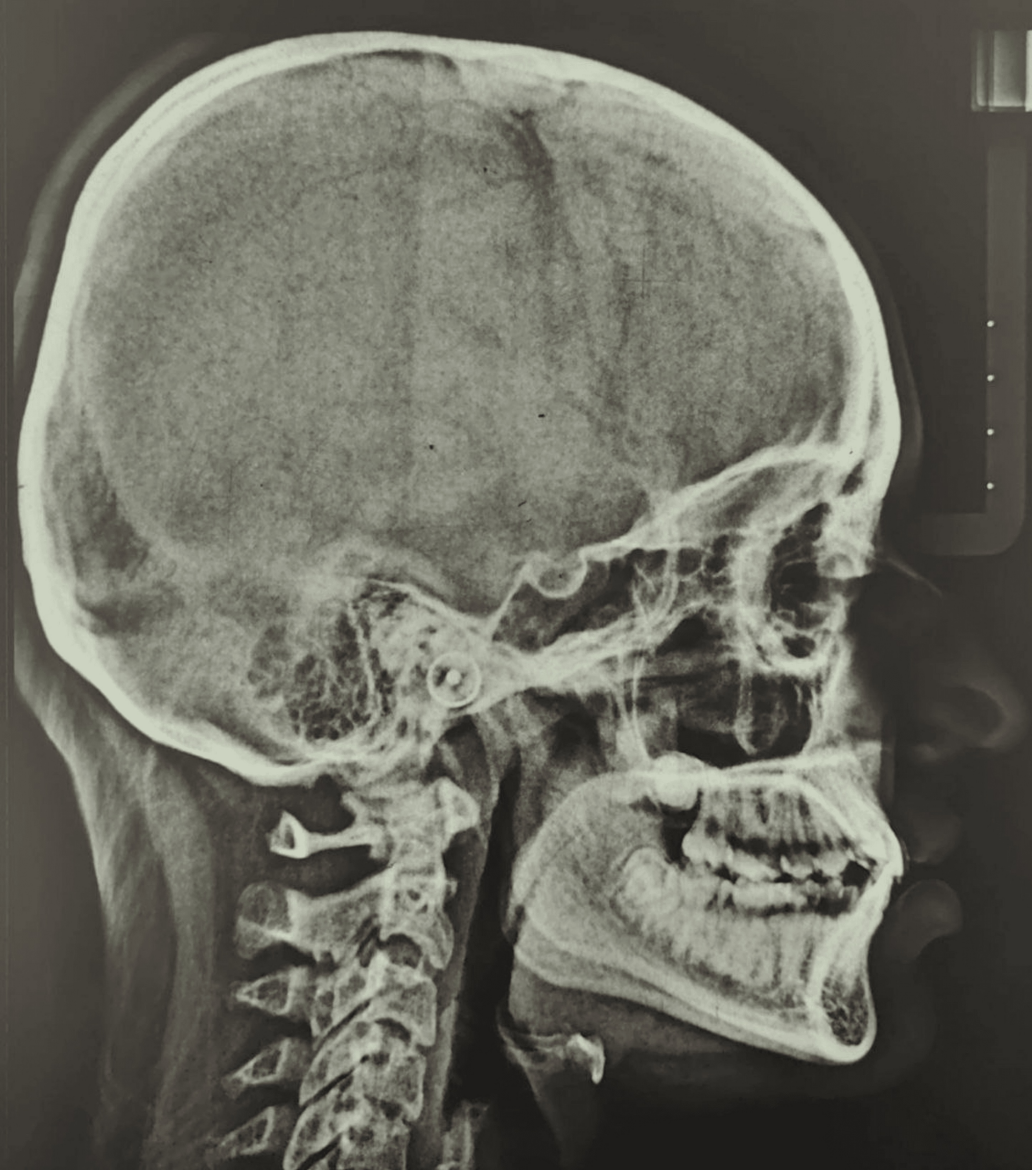
Pretreatment lateral cephalogram

Cephalometric analysis revealed a class I skeletal base with an orthognathic maxilla and mandible. Upper and lower anteriors were proclined (Figure 4).

Results of a cephalometric analysis by Steniers skeletal and dental methods are listed in Table 1. 

**Table 1:** Pre and post-treatment cephalometric values SNA: Sella nasion point A; SNB: Sella nasion point B; ANB: Point A nasion point B; FMA: Frankfort mandibular plane angle; SN: Sella nasion point; NA: Nasion point A; UI: upper incisor; LI: lower incisor; NB: Nasion point B; IMPA: incisor mandibular plane angle

Variables	Mean	Pre-treatment values	Post-treatment values	Difference
Maxilla to cranium				
SNA angle	82±2°	82°	82°	0
Mandible to cranium				
SNB angle	80±2°	80°	80°	0
Maxilla to mandible				
ANB angle	2±2°	2°	2°	0
Wits (mm)	0	2 mm	2 mm	0
Vertical relationship				
Y-axis angle	53-66°	59°	59°	0
Facial axis angle	90°	89°	89°	0
FMA angle	25°	25°	25°	0
Occlusal to SN	23°	22°	22°	0
Maxillary Dental				
UI to NA (angle)	22°	30°	24°	6°
UI to NA (mm)	4 mm	4 mm	4 mm	0 mm
UI to SN (angle)	102±2°	113°	106°	7°
Mandibular dental				
LI to NB (angle)	25°	29°	25°	4°
LI to NB (mm)	4 mm	4 mm	4 mm	0 mm
IMPA (angle)	90±5°	92°	90°	2°
Maxilla to Mandible (dental)				
UI to LI (angle)	130°	118°	126°	-8°

Treatment objectives 

Treatment aimed to achieve a proper alignment of teeth, extraction of mesiodens, closure of spaces present, correction of proclination of incisors, and rehabilitation of the missing lateral incisor. 

Treatment alternatives

Two treatment options were presented to the patient. The first involved the extraction of mesiodens and creating space for the missing lateral incisors bilaterally. The second quadrant would entail the extraction of 28 and subsequent distalisation of the upper left posterior teeth. The estimated treatment time would be 32 months since distalisation could take about five months in itself. 

The second treatment plan required the creation of space for 12 followed by placement of dental implant while going for canine substitution in the second quadrant where the canine would replace the missing upper left lateral incisor. This treatment plan was predicted to be achievable in 24 months. The patient chose the second plan due to aesthetic and psychosocial concerns. 

Treatment progress

**Figure 5:**
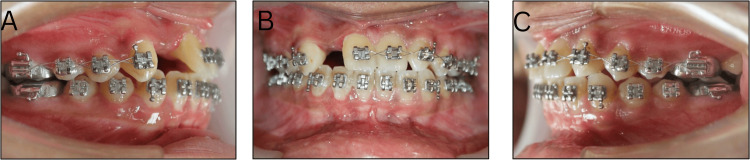
Intraoral photographs before implant placement: (A) Right lateral, (B) Frontal, and (C) Left lateral

Treatment was started by strapping up using the MBT (McLaughlin, Bennett, and Trevisi) 0.022’’ slot bracket system. Initial leveling and alignment of the arches were done with 0.014’’ nickel-titanium (NiTi), 0.016 NiTi followed by 0.016’’ Australian wire in the upper and lower arch. Mesiodens extraction was planned post leveling and alignment of the arches. This was done to preserve the esthetics of the patient and maintain and positive psychosocial impact. Post achievement of optimal alignment of the arches, extraction of the mesiodens was carried out by an oral surgeon. Closure of midline diastema was initiated on 0.018 Australian special plus wire by using a continuous elastic chain engaged between brackets of 11-21 and consolidation of the second quadrant. Closure of the midline diastema was done and optimal space was created for the placement of dental implant in the site of 12. On the right side, a class I molar relationship was maintained and an Angle Class I canine relation was obtained on the right side. On the left side, Angle's Class II canine and molar relationship were maintained (Figure 5). 

**Figure 6:**
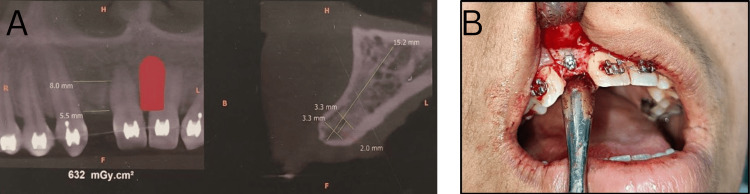
(A) cone-beam CT of 12 area, (B) Dental implant placement for 12

A cone-beam CT (CBCT) of the region was taken to find the bone width in the area of 12 and check its proximity to the surrounding structures. A maxillary surgical stent was prepared for the correct implant position. A mucoperiosteal flap was raised and a dental implant (MAX 2.5; Biocon Limited, Bengaluru, India) was placed by an oral surgeon followed by the placement of a cover screw post in which flap closure was done (Figure 6). During the healing period, the patient was given a riding pontic, a temporary acrylic denture used during fixed orthodontic treatment. The provisional restoration was bonded with a bracket and attached to the main archwire ensuring its stability. 

**Figure 7:**
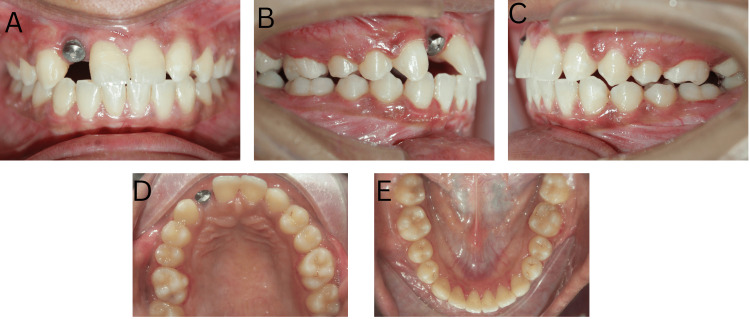
Post-treatment intraoral photographs: (A) Frontal, (B) Right lateral, (C) Left lateral, (D) Maxillary occlusal, and (E) Mandibular occlusal

After three months, the cover screw was removed with the placement of gingival formers. During this period, settling was started and the case was debonded with the placement of a permanent retainer from 11 to 23. The active treatment time was 24 months (Figure 7).

**Figure 8:**
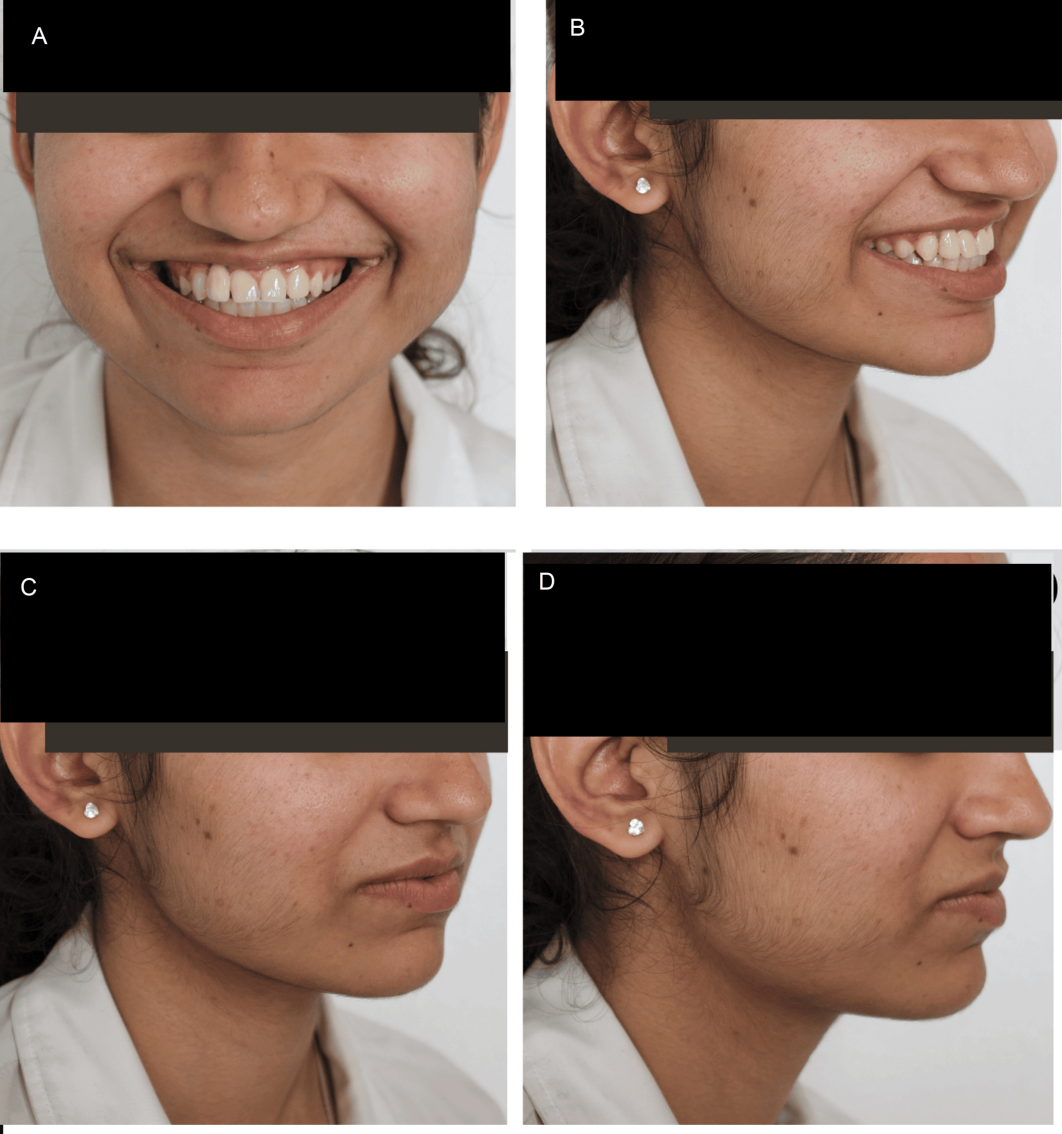
Post-treatment extraoral photographs: (A) Smiling, (B) Smiling 3/4th, (C) 3/4th, and (D) Lateral

Post-debonding a final impression of the maxillary arch was made using vinyl polysiloxane impression material post in which a temporary crown was cemented for two weeks. A final PFM (porcelain fused to metal) crown was placed completing the rehabilitation of the missing 12. To achieve the planned canine substitution in the second quadrant, recontouring of 23 was carried out and the left maxillary canine was reshaped to resemble the contralateral maxillary lateral implant prosthesis. Post-treatment photographs taken six months post completion of orthodontic treatment depict a significant improvement in the patient's smile and confidence (Figure 8).

After the completion of treatment, the patient's chief complaint of an extra tooth and spacing was addressed. The following goals were accomplished during the treatment: alignment of the maxillary and mandibular teeth, removal of mesiodens, closure of anterior spacing, rehabilitation of missing right lateral incisor, and achieving optimal aesthetics with the substitution, and recontouring of 23 to replace the missing left maxillary lateral incisor. A normal overjet and overbite were achieved with Angle's Class II subdivision molar relationship and Class I canine relationship bilaterally.

## Discussion

CHH is considered an anomaly of extremes. The etiology behind CHH remains undetermined. Some reports indicate that the incidence of this anomaly is affected by the combination of genetic and environmental factors. Non-syndromic CHH is a rare occurrence with an incidence of 0.3% in the general population [6]. 

According to Gibson, CHH could be classified as a premaxillary, maxillary, mandibular, and bimaxillary subdivision. However, no definitive classification has been proposed [7]. In the present case, a combination of missing lateral incisors and the presence of mesiodens could be defined as “premaxillary hypo-hyperdontia”. Anthonappa et al reported that CHH does not usually manifest in the same arch and rarely in the same area of the arch [8]. The presented patient displayed the rare occurrence of CHH in the same arch and area of the arch. Treatment options for missing lateral incisors include replacement with a dental implant, canine substitution, and a tooth-supported restoration. Since the presented patient showed bilateral congenitally missing lateral incisors, the decision was made to go ahead with a single tooth implant in the first quadrant and canine substitution in the second quadrant. 

This helped us achieve a Class I relation on the right side with a good cusp to fossa relation on the left side and ending the molar relation in a Class II subdivision. Similarly, Guilherme et al reported a case of missing right lateral incisor with Angle's Class II subdivision [9]. The case was treated while maintaining the Class II subdivision and substituting the right maxillary canine in place of the missing lateral incisor. The treatment choice should be the least invasive and satisfies the expected esthetic and functional goals. Canine substitution can be an ideal option for replacing missing laterals [10]. However, it requires careful diagnosis and treatment planning. Angle's Class II or Class I malocclusions are ideal for canine substitution with a balanced, relatively straight profile. 

Currently, there is no standard treatment protocol for the treatment of CHH, making it a challenging case. Proper diagnosis allows an orthodontist to implement the most appropriate treatment plan for an ideal management

## Conclusions

CHH is a rare condition that requires careful evaluation of all treatment options. Awareness of this rare occurrence allows the clinician to make the correct diagnosis and formulate the right treatment plan. The treatment plan should aim to address the esthetic and functional concerns of the patient.

## References

[1] Rakhshan V (2015). Congenitally missing teeth (hypodontia): A review of the literature concerning the etiology, prevalence, risk factors, patterns and treatment. Dent Res J (Isfahan).

[2] Al-Ani AH, Antoun JS, Thomson WM, Merriman TR, Farella M (2017). Hypodontia: an update on its etiology, classification, and clinical management. Biomed Res Int.

[3] Meighani G, Pakdaman A (2010). Diagnosis and management of supernumerary (mesiodens): a review of the literature. J Dent (Tehran).

[4] Mallineni S.K (2014). Supernumerary teeth: review of the literature with recent updates. Conference Papers in Science.

[5] Solhjou N, Razmjouyi F (2019). Double mesiodens concomitant with missing lateral incisors: a case report. Iran J Ped Dent.

[6] Niranjane P, Tarvade S, Daokar S, Daigavane P, Patil P, Shelke S (2014). Concomitant hypo-hyperdontia- a rare case report. IOSR J Dent Med Sci.

[7] Anthonappa RP, Lee CK, Yiu CK, King NM (2008). Hypohyperdontia: literature review and report of seven cases. Oral Surg Oral Med Oral Pathol Oral Radiol Endod.

[8] Paduano S, Cioffi I, Rongo R, Cupo A, Bucci R, Valletta R (2014). Orthodontic management of congenitally missing maxillary lateral incisors: a case report. Case Rep Dent.

[9] Janson G, Camardella LT, de Freitas MR, de Almeida RR, Martins DR (2009). Treatment of a class II subdivision malocclusion with multiple congenitally missing teeth. Am J Orthod Dentofacial Orthop.

[10] Kokich VO Jr, Kinzer GA (2005). Managing congenitally missing lateral incisors. Part I: canine substitution. J Esthet Restor Dent.

